# Stool substitute transplant therapy for the eradication of *Clostridium difficile* infection: ‘RePOOPulating’ the gut

**DOI:** 10.1186/2049-2618-1-3

**Published:** 2013-01-09

**Authors:** Elaine O Petrof, Gregory B Gloor, Stephen J Vanner, Scott J Weese, David Carter, Michelle C Daigneault, Eric M Brown, Kathleen Schroeter, Emma Allen-Vercoe

**Affiliations:** 1 Department of Medicine, GIDRU Wing, Kingston General Hospital, Queen’s University, 76 Stuart Street, Kingston, ON, K7L 2V7, Canada; 2 Department of Biochemistry, University of Western Ontario, 1151 Richmond Street, London, ON, N6A 3K7, Canada; 3 Department of Pathobiology, University of Guelph, Guelph, 50 Stone Road East, ON, N1G 2W1, Canada; 4 London Regional Genomics Centre, Robarts Research Institute, 100 Perth Drive, London, ON, N6A 5K8, Canada; 5 Department of Molecular and Cellular Biology, University of Guelph, 50 Stone Road East, Guelph, ON, N1G 2W1, Canada

**Keywords:** *Clostridium difficile*, Fecal bacteriotherapy, Probiotics, Gut microbiome

## Abstract

**Background:**

Fecal bacteriotherapy (‘stool transplant’) can be effective in treating recurrent *Clostridium difficile* infection, but concerns of donor infection transmission and patient acceptance limit its use. Here we describe the use of a stool substitute preparation, made from purified intestinal bacterial cultures derived from a single healthy donor, to treat recurrent *C. difficile* infection that had failed repeated standard antibiotics. Thirty-three isolates were recovered from a healthy donor stool sample. Two patients who had failed at least three courses of metronidazole or vancomycin underwent colonoscopy and the mixture was infused throughout the right and mid colon. Pre-treatment and post-treatment stool samples were analyzed by 16 S rRNA gene sequencing using the Ion Torrent platform.

**Results:**

Both patients were infected with the hyper virulent *C. difficile* strain, ribotype 078. Following stool substitute treatment, each patient reverted to their normal bowel pattern within 2 to 3 days and remained symptom-free at 6 months. The analysis demonstrated that rRNA sequences found in the stool substitute were rare in the pre-treatment stool samples but constituted over 25% of the sequences up to 6 months after treatment.

**Conclusion:**

This proof-of-principle study demonstrates that a stool substitute mixture comprising a multi-species community of bacteria is capable of curing antibiotic-resistant *C. difficile* colitis. This benefit correlates with major changes in stool microbial profile and these changes reflect isolates from the synthetic mixture.

**Trial registration:**

Clinical trial registration number: CinicalTrials.gov NCT01372943

## Background

*Clostridium difficile* infection (CDI) is a bacterial disease of the gastrointestinal tract caused by *C. difficile*, a toxin-producing, Gram-positive, anaerobic, spore-forming bacillus. CDI accounts for 15 to 25% of antibiotic-associated diarrhea [1]. The infection occurs most commonly when patients receive antibiotics that alter their normal enteric gut bacteria, allowing overgrowth of *C. difficile*. Recommended therapy for CDI consists of either metronidazole or oral vancomycin [2].

Recurrent CDI is defined as complete resolution of CDI while on appropriate therapy followed by recurrence of CDI after treatment has been stopped [3]. An association has been made between recurrent disease and intestinal dysbiosis [4], and an inability of certain individuals to re-establish their normal intestinal bacteria is thought to play a leading role in recurrence. Unfortunately, few effective treatments exist for those patients with multiple recurrences of CDI, a debilitating disease. Fecal bacteriotherapy, or ‘stool transplant’ – infusing donor stool into the intestine of the recipient to re-establish normal bacterial microbiota – has shown promising results in preliminary studies [3,5] but concerns about pathogen transmission, patient acceptance and inability to standardize the treatment regimen remain. The aim of this study was to determine whether the same disease resolution could be accomplished with a stool substitute. Here we report the successful outcome of two patients with recurrent CDI unresponsive to conventional therapy who received a stool substitute, a preparation of 33 different intestinal bacteria isolated in pure culture, from a single healthy donor.

## Methods

### Study design

The study protocol, in accordance with Good Clinical Practice Guidelines, was approved by the Human Research Ethics Boards at Queen’s University and the University of Guelph, and meets the provisions of the Helsinki Declaration (1964, amended in 2008) of the World Medical Association.

Inclusion criteria for the study included a history of previous CDI, confirmed by *C. difficile* fecal toxin immunoassay, new onset of symptoms after completing a full course of medication for CDI, positive *C. difficile* toxin assay confirming recurrent CDI, and age 18 years or older. Patients were assessed by specialists in Infectious Disease and Gastroenterology, and other possible causes of diarrhea were ruled out. Two patients who fulfilled the inclusion criteria were enrolled in the study and written informed consent was obtained.

### Microbiology

#### Development and preparation of RePOOPulate

The RePOOPulate human probiotic or synthetic stool mixture was developed by extensively culturing the microbial diversity from the stool of a healthy, 41-year-old female donor. Sixty-two different bacterial isolates were recovered on various media types (including Brain Heart Infusion agar, Wilkins–Chalgren agar, Reinforced Clostridial Agar, and deMan, Rogosa & Sharpe agar) using strict anaerobic conditions (to recover both strict and facultative anaerobes). Purified isolates were identified by 16S rRNA gene sequencing and were subjected to antibiotic susceptibility profiling. Susceptibility to antimicrobials was determined either by directly measuring susceptibility or through inference based on other cultivated representatives. For instance, in cases where minimum inhibitory concentration breakpoints are not documented, susceptibility was determined using Kirby–Bauer discs for select antibiotics known to have anaerobic activity; if the bacterial lawn grew up to the edge of the disc, then it was considered resistant and that isolate was not used. For isolates where there was a zone of inhibition of questionable significance, an acceptable level of inhibition was inferred based on other cultivated representatives. If there was any doubt, and the organism was at all suspected to be resistant, then it was not used in the mixture.

Thirty-three isolates, representing commensal species that were generally sensitive to a range of antimicrobials and were relatively straightforward to culture, were selected for the final stool substitute formulation. Full-length 16 S rRNA sequences were aligned with the NAST server [6], and were then classified using the GreenGenes classification server [7]. The most specific name in the GreenGenes classification was used and we report the DNA maximum likelihood score for each classification (Table 1).

**Table 1:** Composition of stool substitute (RePOOPulate)

**Closest species match, inferred by alignment of 16S rRNA sequence to GreenGenes database**^**a**^	**% identity to closest match**	**Relative abundance (by biomass) in RePOOPulate formulation**
*Acidaminococcus intestinalis*	100	+++
*Bacteroides ovatus*	99.52	+
*Bifidobacterium adolescentis* (two different strains)	99.79	++
	99.79	++
*Bifidobacterium longum* (two different strains)	99.86	+++
	99.16	+++
*Blautia producta*	96.43	+
*Clostridium cocleatum*	91.92	+
*Collinsella aerofaciens*	98.73	+
*Dorea longicatena* (two different strains)	99.62	+
	99.60	+
*Escherichia coli*	99.80	+
*Eubacterium desmolans*	94.90	+
*Eubacterium eligens*	98.15	+++++
*Eubacterium limosum*	97.05	+
*Eubacterium rectale* (four different strains)	99.59	+++++
	99.60	+++++
	99.19	+++++
	99.53	+++++
*Eubacterium ventriosum*	100	++
*Faecalibacterium prausnitzii*	99.17	+++++
*Lachnospira pectinoshiza*	95.22	+
*Lactobacillus casei/paracasei*	99.47	+
*Lactobacillus casei*	99.74	+
*Parabacteroides distasonis*	99.45	++
*Raoultella* sp*.*	99.40	+
*Roseburia faecalis*	99.65	++
*Roseburia intestinalis*	100	++
*Ruminococcus torques* (two different strains)	99.15	+++
	99.29	+++
*Ruminococcus obeum* (two different strains)	94.89	+
	94.69	+
*Streptococcus mitis*^b^	99.79	+

List of cultured isolates from the healthy donor, with favorable antibiotic resistance profiles (defined as vancomycin and/or imipenem sensitive, with further sensitivity to at least three of piperacillin, amoxicillin/clavulanic acid, ceftazidime, ceftriaxone, moxifloxacin and metronidazole) that were included in the stool substitute preparation. ^a^Closest species match was inferred by alignment of the 16S rRNA sequence to the GreenGenes database [7]; note that in some cases 16S rRNA gene sequences could not resolve identity beyond genus, and that closest match does not infer definitive speciation. Shaded boxes indicate strains that are possibly novel species (and, in some cases, genera). Note that some representative strains identify with the same species by 16S rRNA gene sequence alignment, but we believe them to be different strains based on differences in colony morphology, antibiotic resistance patterns and growth rates. ^b^Identifies with *S. mitis* but is not β-hemolytic.

To infer a relative ratio of the selected isolates for inclusion in the formulated RePOOPulate product, a comparison of our list of cultured bacterial species was made with the MetaREP metagenomic database collection of stool sample datasets from healthy donors [8]. Using the taxonomy browser, the dataset that most closely matched our profile of cultured isolates (SRS058723) was selected and used as a guide for inference of relative abundance of each species – with the exception that *Bifidobacterium* spp. were added to higher abundances, reflecting the widely observed underestimated abundances of Actinobacteria, and specifically this genus, in metagenomic analyses of human stool [9,10]. An approximate ratio based on cultured cell biomass, measured using standard 10 μl microbiological loops, was generated (Table 1).

Each of the 33 isolates was individually cultured on Fastidious Anaerobe Agar (Lab M Ltd. Heywood, Lancashire, UK) under anaerobic conditions, and then cultures were formulated into the predetermined ratio, as described above, in 100 ml pre-reduced sterile 0.9% normal saline to an estimated concentration of 3.5 × 10^9^ colony-forming units/ml. The bacterial suspension was placed in a reduced atmosphere in a double-sealed container at 4°C, and used within 24 hours of preparation.

#### *Isolation and ribotyping of* C. difficile *from patient stool samples*

*C. difficile* was isolated from stool samples according to methods described previously [11], using selective media of moxalactam norfloxacin broth (CDMN; Oxoid, Nepean, Ontario, Canada) enriched with 0.1% sodium taurocholate. Isolates were typed using the PCR ribotyping method described by Bidet and colleagues [12].

### Administration of stool substitute

Antibiotic therapy was withheld for 2 days and the patients underwent standard colon cleansing the evening prior to colonoscopy. The following morning during colonoscopy, one-half (50 ml) of the solution was deposited in the region of the cecum/proximal ascending colon and the other half was drizzled throughout the transverse colon as the colonoscope was withdrawn. Both patients were noted to have significant diverticular disease. Immediately post procedure, patients were maintained in the Trendelenburg position for 60 minutes before being discharged home. Patients were instructed to eat a fiber-rich diet and not to consume products containing probiotics. Patients were followed by a study nurse to obtain stool samples and closely monitor their clinical response.

### Sequence analysis

#### gDNA extraction from stool samples

gDNA was extracted using a protocol involving a combination of bead beating, the E.Z.N.A.® Stool DNA Kit (Omega Bio-Tek, Norcross, Georgia, USA) and the Maxwell® 16 DNA Purification Kit (Promega, Madison, Wisconsin, USA). Briefly, 200 μl stool sample, 300 μl E.Z.N.A. Kit SLX buffer, 10 μl of 20 mg/ml proteinase K (in 0.1 mM CaCl_2_) and 200 mg glass beads were added to a screw-capped Eppendorf tube and disrupted in a bead beater for 3 minutes. Following subsequent incubation at 70°C for 10 minutes and at 95°C for 2 minutes, 100 μl E.Z.N.A. Kit Buffer P2 was added to each sample and incubated on ice for 5 minutes. Samples were then centrifuged at 14,000 × *g* for 5 minutes, and the supernatant transferred into new tubes, each containing 200 μl E.Z.N.A. Kit HTR reagent. Following thorough mixing, samples were incubated at room temperature for 2 minutes and centrifuged at 14,000 × *g*, and the supernatant was transferred into Maxwell® 16 DNA Purification Kit cartridges. The remainder of the DNA extraction protocol was carried out in the Maxwell® 16 Instrument according to the manufacturer’s instructions (Promega).

#### V6 rRNA amplification

PCR amplification of the bacterial V6 rRNA region was carried out with the left-side primer CWACGCGARGAACCTTACC and the right-side primer ACRACACGAGCTGACGAC. These primer sequences were chosen because they are exact matches to >95% of the rRNA sequences from organisms identified in the human microbiome project (GBG, unpublished observations). In addition the left-side primers contained the standard Ion Torrent (Ion Torrent Systems Inc., Guilford, Connecticut, USA) adapter and key sequence at their 5^′^ end (CCATCTCATCCCTGCGTGTCTCCGACTCAG). One of the following 5-mer barcodes was located between the 3^′^ end of the key sequence and the 5^′^ end of the primer: TATCG, TAGAC, TGCAT, ATGAG, ACAGT, AGATG, CTCAC, CTGTA, CGTGA, CGACT, AACTC, or CCTAT. Duplicate samples did not use the same barcodes. The right-side primer had the other standard Ion Torrent adapter sequence (CCTCTCTATGGGCAGTCGGTGAT) attached to its 5^′^ end. Amplification was performed for 25 cycles in 40 μl using the colorless GO-Taq hot start master mix (Promega) according to the manufacturer's instructions with the following three-step temperature profile: 95°C, 55°C and 72°C for 1 minute each step, then 5 μl of the resulting amplification were quantified using the QuBit broad-range double-stranded DNA fluorometric quantitation reagent (InVitroGen, Life technologies Inc., Burlington, Ontario, Canada). Samples were pooled at approximately equal concentrations and purified using a Wizard PCR Clean-Up Kit (Promega).

#### Sequencing

Sequence reactions were carried out on the Ion Torrent 314 and 316 chip platform. Up to 12 samples were multiplexed on each chip through use of individual sequence tags. Data from all runs were pooled when samples were run on more than one chip.

#### Sequence data processing

Five sequencing reactions were carried out on the Ion Torrent platform: three reactions on a 314 chip and two reactions on the 316 chip. The chips differ only in the density of the spots, and hence in the amount of sequence that can be obtained (the 316 chip is about five to six times as dense as the 314 chip). The sequence was provided in fastq format. All sequences were then filtered according to the following criteria: exact matches to the barcodes used, exact match to the left-side primer including redundant positions in the primer, an exact match to the first six nucleotides of the right-side primer, and a length between the left-side and right-side primer of between 71 and 83 nucleotides. This length was chosen because it encompasses the predicted amplicon product size from all human-associated bacterial organisms that have been cultured and sequenced as part of the human microbiome project.

Approximately 40 to 50% of the reads passed these filters in the most recent Ion Torrent runs; reads not passing the filters were not examined further. Reads were processed as described by Gloor and colleagues [13] except that clustering with USEARCH was performed at 97% identity. Chimera detection was performed with UCHIME (version v5.2.32) using the *de novo* method [14]. Only four chimeric sequences were observed out of 30,419 unique sequences in the merged dataset, and all were rare. This frequency is similar to that reported previously for amplification and sequencing of the V6 rRNA region using the Illumina platform [13]. Chimeric sequences were not considered an issue in this dataset.

A table of counts for sequences grouped at the 97% operational taxonomic unit (OTU) and 100% identical sequence unit identity level were generated for each sample as before [13], keeping all identical sequence unit or OTU sequences that were represented in any sample at a frequency >0.5%. Reads that were never abundant in any sample (<0.5%) were grouped into the remainder and discarded. Between 12.6 and 51.9% (median 31%) of the identical sequence unit reads and between 1.4 and 17.2% (median 5.8%) of the OTU reads were in the remainder group. These values are approximately five times greater than those observed for identical sequence units sequenced on the Illumina platform but are about equivalent to the Illumina platform observations when reads were clustered.

#### Taxonomic classification

Classification of the sequences by either the GreenGenes or RDP classifiers proved to be unreliable because of the short length of the V6 region. Classification of the sequences present in the count table was therefore performed using the RDP closest match option on the full-length, high-quality, isolated subset. The maximum number of best hits was identified, and the taxonomic classification of the best match and ties was collected. The classification of those hits was adopted for all levels where the classification was identical across all best matches, otherwise the classification was marked as undefined. The V6 region is not able to resolve the genus or species level of a number of clades, so all analyses were carried out at the family level. This strategy worked for all abundant families – with the exception of the *Bifidobacterium*, which were annotated as such from BLAST searches of the NCBI microbial 16 S rRNA database. The taxonomic classification was added to the sequence count table and the data were presented in formats that could be accepted by QIIME 1.5.0 [15] as follows. Sequence alignments were built using Muscle [16] and a neighbor-joining tree was generated by ClustalW2 [17]. Beta-diversity was calculated by the UniFrac algorithm [18]. Tables were imported into MacQIIME, which is an OS X bundled version of QIIME 1.5.0, and were analyzed using the default parameters.

## Results

### Clinical outcomes

#### Patient 1

**Figure 1:**
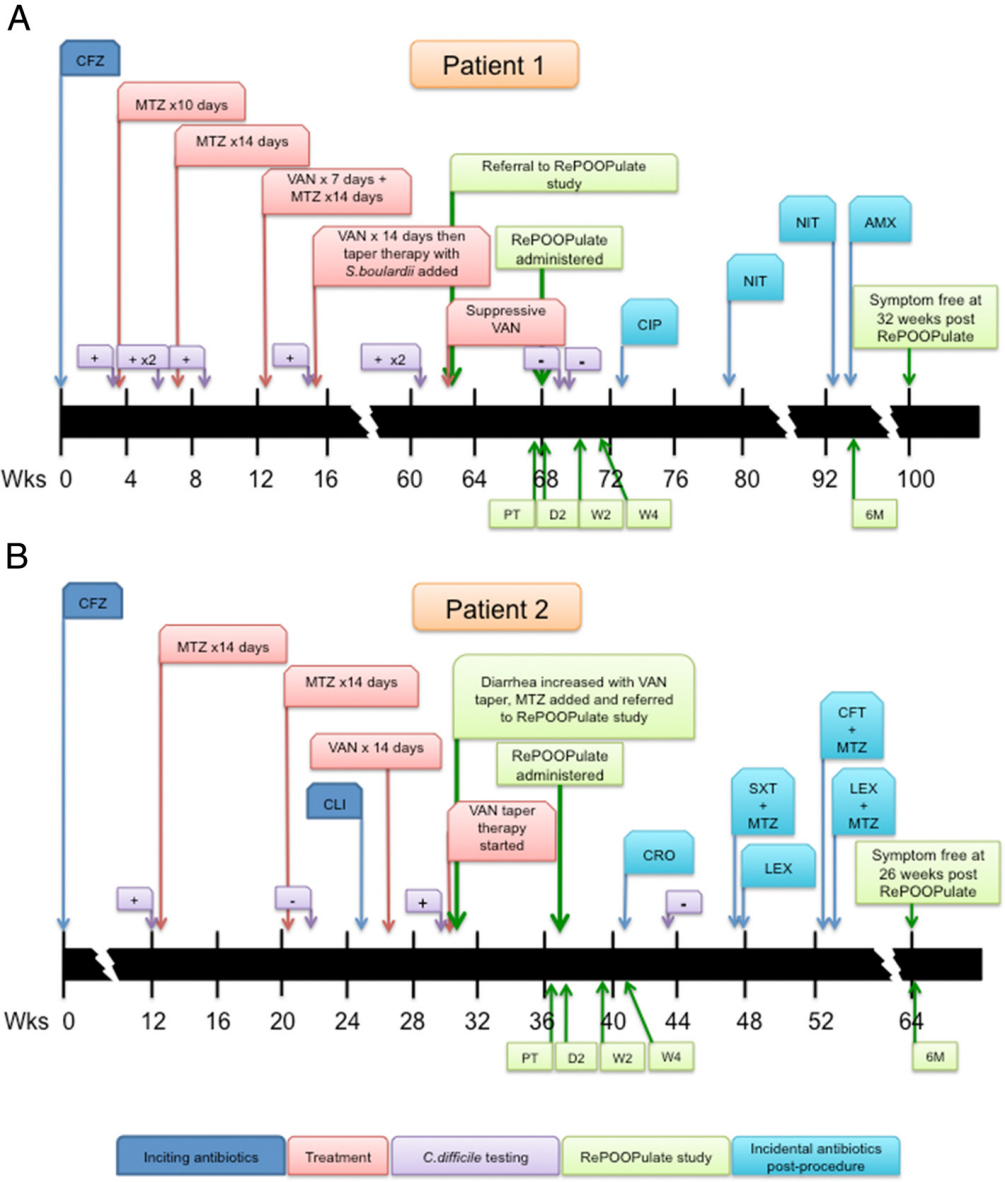
**Clinical timeline of events for Patients 1 and 2.** Sequence of events for the first two patients enrolled in the study. **(A)** Patient 1 had *Clostridium difficile* initially occurring after a pre-operative course of cefazolin for elective total knee arthroplasty. **(B)** Patient 2 had *C. difficile* initially occurring after a course of cefazolin for cellulitis. Both patients had multiple courses of antibiotic treatment for the *C. difficile* infection with both vancomycin and metronidazole prior to enrollment, as indicated. In addition, Patient 1 received the probiotic *Saccharomyces boulardii*. Prior to treatment with the stool substitute preparation RePOOPulate (RP), stool collection on each patient was carried out at 2 days pre treatment (PT), day 2 post treatment (D2), week 2 post treatment (W2), week 4 post treatment (W4), and 6 months post treatment (6 M). Toxin assays for *C. difficile* were also performed (purple boxes), with results as shown. Incidental antibiotic use post treatment is indicated. AMX, amoxicillin; CFZ, cefazolin; CIP, ciprofloxacin; CLI, clindamycin; CRO, ceftriaxone; LEX, cephalexin; MTZ, metronidazole; NIT, nitrofurantoin; SXT, trimethoprim-sulfamethoxazole; VAN, vancomycin

Patient 1 was a 74-year-old Caucasian woman who presented with six episodes of recurrent CDI (confirmed by *C. difficile* toxin assay) over an 18-month period, all of which required hospitalization. The first episode of CDI developed after elective orthopedic surgery (knee arthroplasty), when the patient received preoperative cefazolin (Figure 1A). After treatment with the stool substitute, she reverted to her normal bowel pattern of a formed stool every 1 or 2 days. No *C. difficile* was detectable by *C. difficile* toxin assay at 10 days post procedure. Patient 1 did receive several courses of antibiotics for recurrent urinary tract infections in the subsequent weeks following her stool substitute treatment, but her diarrhea did not recur. She remained symptom-free at the last evaluation, 24 weeks after treatment. Two different strains of *C. difficile* were isolated from the pre-treatment sample. One strain was identified as ribotype 078; the other was a less common toxinotype 0 ribotype (data not shown).

#### Patient 2

Patient 2 was a 70-year-old Caucasian woman with a history of peripheral neuropathy, which predisposed her to recurrent skin and soft tissue infections. She developed her initial CDI after receiving cefazolin for cellulitis and presented to the clinic with a history of three episodes of recurrent CDI, the last of which had failed standard medical therapy (Figure 1B). After receiving the study treatment, she reported formed bowel movements within 72 hours. She remained symptom-free for 3 weeks, then developed recurrent cellulitis and was placed on ceftriaxone by her physician. She was monitored closely while on ceftriaxone but did not develop loose stool or diarrhea. She suffered from several skin and soft tissue infections in the subsequent weeks and received several additional courses of broad-spectrum antibiotics for these infections. Nevertheless, she remained symptom-free with no diarrhea at last evaluation, which was 26 weeks post procedure. Ribotype 078 was also isolated from this patient’s pre-treatment sample.

### Sequence analysis

#### Reproducibility of the data

The Ion Torrent instrument has not previously been used for community microbial composition analysis with amplified rRNA variable regions. We therefore first examined the reproducibility of the reads obtained on the instrument by performing three separate PCR amplifications of the V6 rRNA region and sequencing these amplifications on four separate Ion Torrent runs. The PCR reactions were amplified by two separate individuals on separate days. A separate library was prepared from each amplification. Each library was run on either a 314 or a 316 Ion Torrent chip, with one library run on two separate chips. In this way the technical replication both of the amplification and of the sequencing reaction could be assessed.

**Figure 2:**
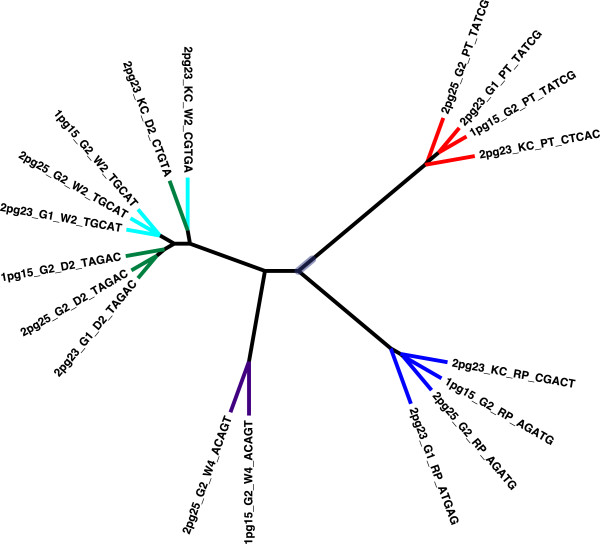
**Distance tree of weighted UniFrac distances between samples for Patient 1 amplified and sequenced independently.** Distance tree calculated by the unweighted pair group method with arithmetic mean. Branch tips are colored by sample: red, pre-treatment; blue, RePOOPulate formulation. Post-treatment samples are colored green (D2), cyan (W2), and purple (W4). Tip label fields are separated by an underscore character and the fields are: Ion Torrent run ID, person and time of amplification, sample identifier, barcode sequence

The number of reads obtained for these sequencing reactions was often small – especially for the initial run on the 314 chip, which has limited capacity – and is summarized in Table 2. Reads were processed by the standard pipeline outlined in Methods, and an unweighted pair group method with arithmetic mean distance tree was generated from the beta-diversity output by QIIME. The result (shown in Figure 2) demonstrates that all the amplifications from each of the four replicates clustered together by sample – with the exception of one of the Patient 1 day 2 and week 2 samples, which showed clustering together by the amplification. Note, however, the very short branch lengths in this clade, indicating that these samples are probably indistinguishable. All further analyses used pooled reads across all replicates for each sample.

**Table 2:** Read numbers for sequencing reactions obtained on the Ion Torrent platform

**Run ID**	**Person**	**Barcode**	**Sample**	**Reads**
1 pg15	G2	TAGAC	D2	1,568
1 pg15	G2	TATCG	PT	1,058
1 pg15	G2	AGATG	RP	1,439
1 pg15	G2	TGCAT	W2	1,927
1 pg15	G2	ACAGT	W4	1,319
2 pg23	G1	TAGAC	D2	782
2 pg23	G1	TATCG	PT	655
2 pg23	G1	ATGAG	RP	920
2 pg23	G1	TGCAT	W2	981
2 pg23	KC	CTGTA	D2	2,506
2 pg23	KC	CTCAC	PT	524
2 pg23	KC	CGACT	RP	702
2 pg23	KC	CGTGA	W2	3,519
2 pg25	G2	TAGAC	D2	1,082
2 pg25	G2	TATCG	PT	532
2 pg25	G2	AGATG	RP	526
2 pg25	G2	TGCAT	W2	925
2 pg25	G2	ACAGT	W4	563

*D2*, day 2 post treatment; *PT*, 2 days pre treatment; *RP*, RePOOPulate preparation; *W2*, week 2 post treatment; *W4*, week 4 post treatment.

#### Examination of alpha-diversity

In total, there were between 3,758 and 76,752 V6 rRNA reads per sample for Patient 1 and between 19,751 and 64,200 reads per sample for Patient 2 using the Ion Torrent instrument as outlined in Methods. These reads were processed by a combination of custom scripts and the QIIME pipeline as described in Methods. Reads were clustered at 97% sequence identity for the analysis that follows, unless stated otherwise. Read counts were normalized using rarefaction to the minimum number of reads per sample in each patient, and Shannon's diversity index was plotted for each intermediate rarefaction level and for the nonrarefied data. We observed that the mean Shannon's diversity index of 10 rarefaction samples approximated the diversity index of the total dataset when the number of rarefied samples exceeded 1,000 (data not shown). This observation indicates that we obtained sufficient reads in all samples to accurately estimate the diversity. Shannon's diversity on the total dataset for all samples is given in Table 3, from which we see that the two patients had dramatically different Shannon's diversity scores before and after treatment. Patient 1 had a highly diverse microbiota that became less diverse after treatment, and over time tended to become more diverse. At 6 months post treatment, this patient had a diversity score that was almost the same as that at pre treatment. Patient 2 initially had a low diversity microbiota, which became more diverse following treatment and stabilized over the long term at a level that was more diverse than that at pre treatment.

**Table 3:** Shannon diversity values calculated for nonrarefied count values

**Patient**	**PT**	**D2**	**W2**	**W4**	**6 M**	**RP**
1	5.1	3.1	3.4	5.0	5.2	4.1
2	3.2	3.5	4.2	3.8	3.9	4.1

*D2*, day 2 post treatment; 6 M, 6 months post treatment; *PT*, 2 days pre treatment; *RP*, RePOOPulate preparation; *W2*, week 2 post treatment; *W4*, week 4 post treatment.

#### Examination of beta-diversity

**Figure 3:**
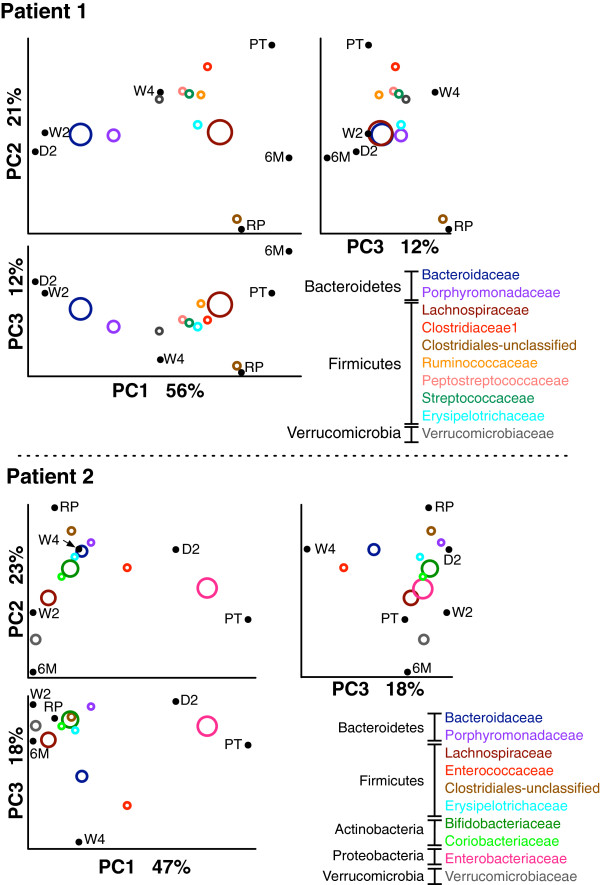
**Principle component coordinates of patient time points and most abundant sequences clustered at family level.** Weighted UniFrac principle coordinates were generated by QIIME for each patient independently. These time points are denoted PT for pre treatment, RP for the RePOOPulate formula, and as the day (D), week (W) or month (M) time point post treatment. The weighted mean abundance of family-level taxonomic groups is indicated by the size and position of the open circles. For example, for Patient 1 Bacteriodaceae are abundant in the day 2 and week 2 post-treatment samples, less abundant in the week 4 post-treatment sample, and are rare in all other samples. Only the 10 most abundant groupings of organisms are shown, and these differ between the two patients, although the Lachnospiraceae family is abundant in both

Taxonomic assignments of the seed sequences for each OTU were derived from best-hit analysis of the sequences in the RDP database as explained in Methods. Briefly, the full taxonomic lineage of the 20 best hits and ties was captured using a custom Perl script and added to the QIIME input tables. Any lineage where the best hits and ties were not in full agreement was annotated as undefined. Taxonomic assignment was carried out to the family level since the rRNA V6 region has poor resolution below this taxonomic level for several groups found in our dataset, such as the Gammaproteobacteria and Lachnospiraceae families. Beta-diversity taxonomic bi-plots at the family level were generated using the QIIME package with default values for the read counts of the samples derived from each individual patient including the initial RePOOPulate sample (Figure 3). In both patients, the first three principle components captured over 85% of the variation between the samples.

**Figure 4:**
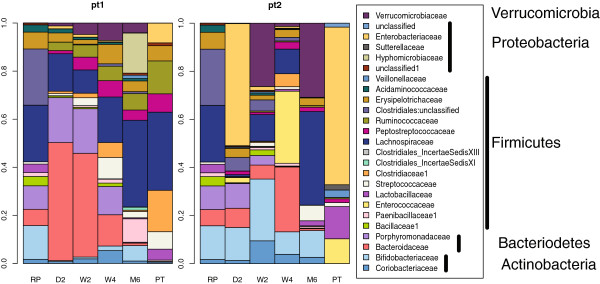
**Barplot of abundance at the family level.** Operational taxonomic units (OTUs) that comprised more than 0.5% of the OTUs in any sample were grouped into the appropriate family and plotted. These plots show how the actual composition of each sample changes over time. Note that the two patients had very different initial microbiota compositions. The compositional differences were maintained at all time points, suggesting that environmental or genetic factors were important in shaping community structure

The taxonomic distribution of reads in the two patients was noticeably different, however, as shown in the barplots of Figure 4 – as was the trajectory of the microbiome composition after treatment. The microbiota of Patient 1 initially had a number of distinct families from the Firmicutes phylum. Samples collected at day 2 and at week 2 were largely composed of families from the Bacteriodetes. However, samples collected after 4 weeks were composed of similar fractions of families in these two phyla. After 6 months this patient had a microbiota that was largely composed of Firmicutes. In contrast, the microbiota of Patient 2 was largely composed of Proteobacteria before treatment, and was noticeably lacking in Actinobacteria and Bacteriodetes phyla. The fraction of Proteobacteria declined rapidly after treatment, initially displaced by families from the Bacteriodetes and Actinobacteria. At later time points (2 to 4 weeks post treatment) there was a reduction of Actinobacteria and an increase in Bacteriodetes and Firmicutes. The Proteobacteria were displaced completely by 2 weeks. After 6 months this patient’s microbiota was composed largely of families drawn from the Firmicutes and of Verrucomicrobia phyla.

#### Long-term colonization

**Figure 5:**
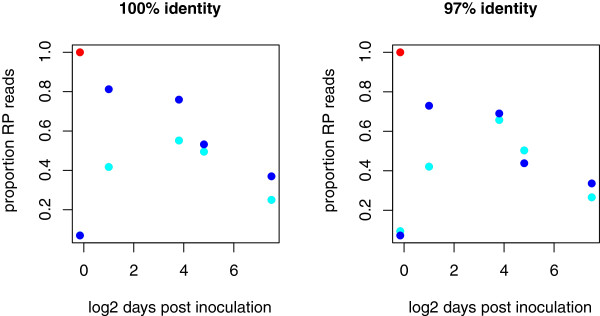
**Weighted abundance overlap at the identical sequence unit and 97%-clustered operational taxonomic unit levels.** Proportion of sequence counts that correspond exactly to those in the RePOOPulate (RP) formulation and found in each patient sample as a function of time post treatment. Red, RP formulation; dark blue, samples from Patient 1; cyan, samples from Patient 2. There is an initial increase in reads identical to the RP reads immediately after treatment, and a steady decline in proportion for each patient with time since treatment. Both patients had similar RP-identical reads at 6 months post treatment, even though their microbiota profiles were different

We were interested in determining the ability of the organisms composing the RePOOPulate formulation to stably colonize the distal colon of the patients. We noted that the weighted UniFrac distances between the samples at pre treatment and 6 months post treatment in Patient 1 were less than those between either sample and any other. In contrast, the earliest time point for Patient 2 was most similar to the pre-treatment sample. These relationships can be seen in Figures 3 and 4, but are clearer in Figure S1 in Additional file 1. However, there was one common feature between the microbiota trajectories of the two patients. Figure 5 shows plots of the weighted fraction of sequences that were identical to the sequences found in the RePOOPulate sample clustered at 97% and 100% identity. Interestingly, even though the initial taxonomic distribution of the patients was very different (Figures 3 and 4), the initial fraction of reads identical to the RePOOPulate reads was <7% when clustered at 100% identity, and was between 7 and 9.5% when clustered at 97% identity. Not surprisingly, the fraction of reads identical to those derived from the RePOOPulate sample increased rapidly after treatment such that reads identical to the RePOOPulate reads 2 days to 2 weeks after treatment composed >70% of the total reads in Patient 1 (day 2 post treatment) and 50% in Patient 2 (week 2 post treatment). The fraction of the patient microbiota that was composed of reads identical to those found in RePOOPulate declined continuously from the sample 2 weeks post treatment onwards, and at 6 months these reads were found to compose between 25 and 36% of the total reads obtained from each patient. This emergent pattern of slow loss of reads identical to reads in the RePOOPulate sample was common to both patients.

## Discussion

This pilot study shows that a synthetic stool (stool substitute) may be an effective and feasible alternative to the use of defecated donor fecal matter (stool transplant) in the treatment of recurrent CDI. Given the marked differences between the two patients in the study, it is difficult to draw conclusions that might be broadly applicable to a larger patient population at this time and clearly more patients are needed. Nevertheless, the clinical cure achieved at 6 months of follow-up demonstrates feasibility of this approach as an alternative to conventional stool transplant. A synthetic stool substitute approach has multiple advantages: the exact composition of bacteria administered is known and can be controlled; the bacterial species composition can be reproduced, should a future treatment be necessary; preparations of pure culture are more stable than stool, which some groups recommend should be collected fresh and instilled into the recipient within 6 hours of collection [19]; an absence of viruses and other pathogens in the administered mixture can be ensured, thereby improving patient safety; and the administered organisms can be selected based on their sensitivity to antimicrobials, allowing an enhanced safety profile.

Recurrent CDI is thought to be largely due to the inability of the normal intestinal microflora to recover and re-establish itself [4,20,21]. We used the Ion Torrent platform to analyze the 16 S rRNA gene profiles of stool samples collected from each patient during the study, and carried out exhaustive quality control of our data. We concluded that this sequencing platform, together with the PCR amplification protocol and bioinformatic analysis pipeline, was adequate to reproducibly separate both technical replicate samples (Figure 2).

Our study showed that the microbiota of both patients adapted characteristics of the stool substitute mixture yet still retained some of their original microbiota, similar to that described for stool transplants [20]. However, our data suggest that decreased diversity as a risk factor for recurrent CDI may be less important than the actual organisms present in the mixture *per se*, since Patient 1 actually had a very diverse microbiome at the outset but still suffered from severe recurrent CDI. Sequences identical to those of the stool substitute bacteria were initially rare in the pre-treatment samples for both patients (<7%), but became transiently abundant and constituted over 25% of the sequences up to 6 months after stool substitute treatment was given. Hence, we conclude that some of the administered bacteria are stably colonizing the colon, an important observation since most commercially available probiotics only transiently colonize the intestine. These data along with the very different set of RePOOPulate organisms that were initially abundant suggest that other factors, such as diet, may play an active role in influencing the microbial communities over time. These data suggest that a multi-species derivative community such as that used here will be more generally useful than a single organism probiotic or a mixed culture of such probiotic species, because the microbes in RePOOPulate are derived from a community and probably retain some community structure that enables them to colonize the appropriate environment. In addition, the data also suggest that the relative proportions of different bacterial strains in the formulation are of only minor importance. Experiments to determine the extent of the influence of therapeutic community composition on the resulting community profile in the host are currently underway. Longer term experiments colonizing animal models with the stool substitute formulations will clarify the extent to which organisms introduced as part of a gut microbial ecosystem are perturbed by antibiotic use, and the extent to which they persist over longer time frames.

The question of whether age-matching of donors and recipients of stool would be of relevance, since the isolates used to formulate our stool substitute were derived from a younger individual, is largely a matter of debate. Many stool transplants have been performed successfully in older patients using young donors that are often related to the patient [22,23]. The treatment seems equally effective when the young donors are not related [19,23]. We therefore suspect that age-matching will not turn out to be a critical factor in treatment success, although this is a question that should be addressed by careful meta-analysis of clinical trial data. Similarly, the relevance of patient enterotype [24], which may turn out to be more important than age-matching, has not been fully explored. Experiments are currently underway in our laboratories to develop further therapeutic synthetic stool mixtures to represent the different enterotypes known to exist in humans.

Both patients were infected with ribotype 078, an emerging hypervirulent strain that has been associated with community-associated CDI, as well as with livestock (pigs, cattle, poultry), and food products [25]. Like NAP1/027, its prevalence seems to be increasing in many countries [25]. Whether ribotype 078 will be more commonly found in patients with recurrent CDI, whether it is an emerging pathogen in this region or whether its presence in these two non-epidemiologically related patients was a chance finding remains to be seen. Regardless, the stool substitute preparation used here was effective at eradicating disease that had failed all other treatment regimens. In addition, this study also suggests that a defined microbial community, isolated from a single healthy donor, may be robust enough to withstand further perturbations by antibiotics as illustrated by the patients in our study. In the case of Patient 1, who suffered from occasional urinary tract infections, the antibiotics used post procedure (ciprofloxacin, nitrofurantoin and amoxicillin) were for short courses only, up to a maximum of 7 days. For Patient 2, her recurrent skin and soft tissue infections occasionally necessitated a broad-spectrum antibiotic combination (for example, cephalexin and metronidazole) of much longer duration (4 weeks in one case). Despite post-procedure administration of these incidental antibiotics for infections unrelated to *C. difficile* colitis, neither patient developed further recurrent CDI. However, at this time it remains unclear whether antibiotic administration affected the long-term colonization by the microbial community used as treatment, or to what extent the differences in microbial profile in the 6-month samples between patients is driven by the different antibiotics administered. More controlled studies in animal models may help to address some of these questions.

## Conclusion

Further research is needed to define the mechanisms and precise role for this stool substitute in treating CDI. However, once a patient fails oral vancomycin therapy, treatment options for recurrent CDI are very limited. Ecosystem therapeutics or repopulating the bowel with defined communities of normal intestinal bacteria offers another effective therapy for treating recurrent CDI.

## Abbreviations

CDI: *Clostridium difficile* infection; gDNA: genomic DNA; OTU: Operational taxonomic unit; PCR: Polymerase chain reaction; QIIME: Quantitative Insights Into Microbial Ecology; V6 rRNA: Variable region 6 of the 16S ribosomal RNA gene.

## Competing interests

EOP, EA-V and SJV have filed a provisional patent together. The remaining authors declare that they have no competing interests.

## Authors' contributions

EOP and GBG contributed equally to this work. EOP and EA-V conceived of the study, developed its design and coordination and drafted the manuscript. DC carried out the sequencing work. GBG carried out the bioinformatic analyses and helped to draft the manuscript. SJV participated in the clinical design and helped to draft the manuscript. JSW cultured *C. difficile* isolates from patients and determined their ribotypes. MCD, EMB and KS isolated, characterized and cultured the stool substitute components, and prepared gDNA for sequencing from patient samples. All authors read and approved the final manuscript.

## Supplementary Material

Additional file 1 **Figure S1.** Unweighted pair group method with arithmetic mean distance tree of the weighted UniFrac distances between samples for Patients 1 and 2. The branch tips are labeled with the sample names for each patient. The scale bar is shown for each patient. Click here for file
