# Supplementary figures and images for: Stool substitute transplant therapy for the eradication of Clostridium difficile infection: ‘RePOOPulating’ the gut
Source: Microbiome. 2013 Jan 9;1:3. doi: 10.1186/2049-2618-1-3 (PMC3869191; doi:10.1186/2049-2618-1-3)

Patient 1

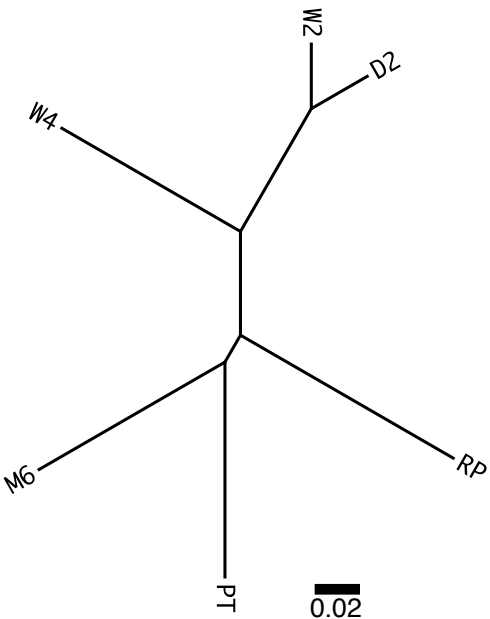

Patient 2

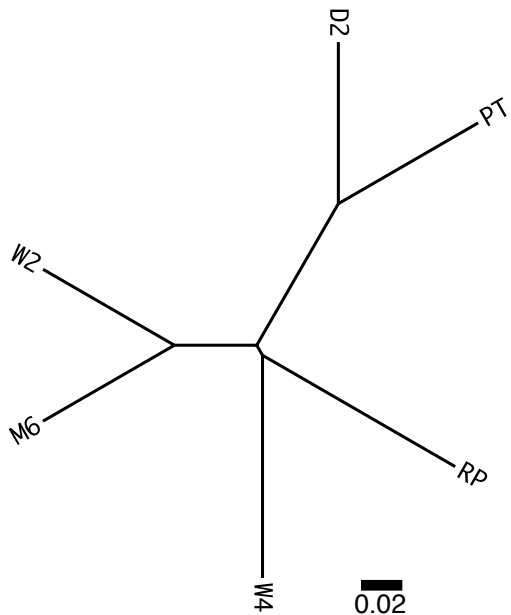

Supplement: Additional file 1 — Figure S1. Unweighted pair group method with arithmetic mean distance tree of the weighted UniFrac distances between samples for Patients 1 and 2. The branch tips are labeled with the sample names for each patient. The scale bar is shown for each patient. [file 2049-2618-1-3-S1.pdf]
